# Peri-ovulatory putrescine to reduce aneuploid conceptions

**DOI:** 10.18632/aging.100500

**Published:** 2012-11-23

**Authors:** X. Johné Liu, Yong Tao

**Affiliations:** ^1^ Ottawa Hospital Research Institute, The Ottawa Hospital - General Campus, 501 Smythe Road, box 511, Ottawa, Ontario, K1H 6X9. Canada; ^2^ Department of Obstetrics and Gynecology, University of Ottawa, Ontario, K1H 6X9, Canada; ^3^ Department of Biochemistry, Microbiology and Immunology, University of Ottawa, Ontario, K1H 6X9, Canada; ^4^ Faculty of Graduate and Postdoctoral Studies, University of Ottawa, Ontario, K1H 6X9, Canada

Women experience diminished fertility, increased risk of miscarriages and congenital birth defects in their late 30s and early 40s, 10-15 years before reaching menopause. Egg aneuploidy (having an incorrect number of chromosomes) is the most important etiology for these reproductive problems in older women. Science correspondent Jon Cohen wrote a News piece in 2002 [1] on the quest to understand the mechanisms of maternal aging-related aneuploidies in humans, which is still applicable today. Although we have made impressive progress in understanding how aneuploidies arise, we know little about providing medical interventions to reduce them. “My Holy Grail is to deliver some sort of aneuploidy-reduction pill, …”, Dr. Terry Hassold, a leading geneticist in the field, is quoted as saying. Our recent study [2] may be a step towards developing such a pill.

**Figure 1:**
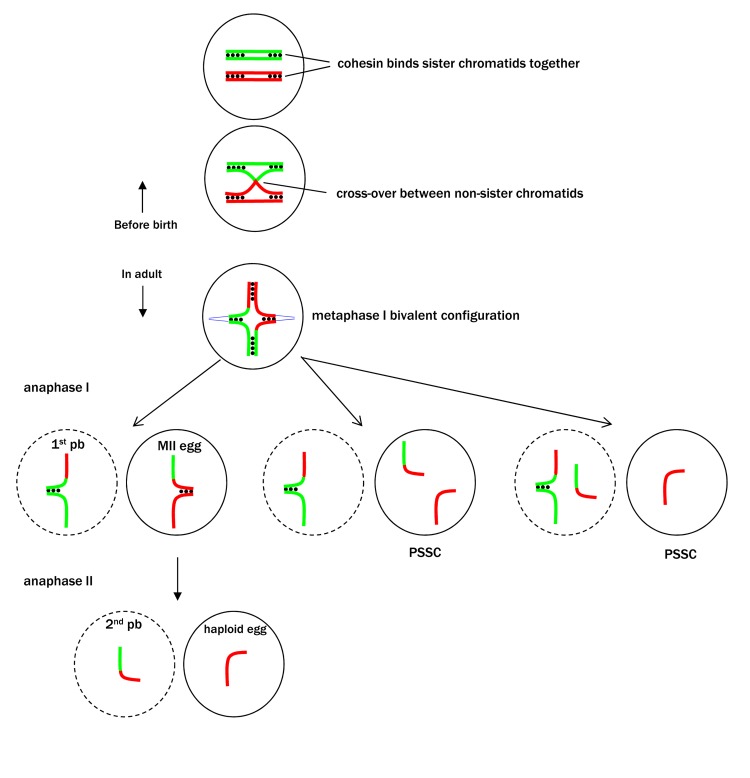
Premature separation of sister chromatids (PSSC) during oocyte maturation During each estrous cycle (menstrual cycle in women), the oocyte undergoes two asymmetric cell divisions (meiosis I and meiosis II), each expelling half of the chromosomes to a non-viable polar body (pb). A chromosome bivalent, consisting of four chromatids, is depicted with the centromeres attached to spindle microtubules (blue lines) at metaphase I. During oocyte maturation (meiosis I), cohesin complexes distal to the cross-over are removed but those close to the centromeres are retained, segregating the sisters together to the egg or the polar body (left scenario). Sister chromatids inadvertently separated in meiosis I could both end up in the egg (middle scenario) or partitioned between the egg and 1^st^ polar body (right scenario). Either way, the egg is considered PSSC. Sister chromatids are separated during fertilization (meiosis II) when chromosome cohesin is completely removed. The schematic representation of mouse chromosomes is adapted from Kudo et al. 2006. Cell. 126:135-146.

Aneuploid eggs result in aneuploid conceptions, most of which are lost either prior to clinical recognition of pregnancies or by miscarriages. The small proportion that survive inevitably carry major birth defects. Accurate egg aneuploidy rates in the general population are not known, but numerous analyses of IVF samples have revealed staggeringly high rates. Notably, a recent study of more than 20,000 IVF eggs revealed significant age-related aneuploidies, reaching >40% in the 40 year old age group [3]. Furthermore, this study [3] employs fluorescence in situ hybridization of polar bodies and is therefore limited in the number of chromosomes (five) that can be analyzed simultaneously. The true aneuploidy rates in older women, considering all 23 chromosomes, would be expected to be much higher. This unusually high egg aneuploidy rate in humans is likely due to a combination of our longevity and the peculiar oogenesis (the process of generating eggs) in all vertebrates including humans. Unlike vertebrate males that continuously generate mature sperm from germ line stem cells through adulthood, in vertebrates oogenesis begins during the embryonic stage when germ line stem cells initiate meiosis and develop into primary oocytes. By birth, the females have developed a finite number of primary oocytes arrested in meiotic prophase that comprises their lifetime egg supply. The prophase oocytes contain chromosome bivalents each having four chromatids linked together by a combination of sister chromotid cohesion and non-sister cross-over (Figure 1), with this linkage persisting for decades in women. During ovulation the two sister chromatids are segregated to the same cell (egg or 1st polar body) in a process called oocyte maturation; in the subsequent fertilization, the sisters are segregated between the 2nd polar body and the haploid egg. As females age, chromosome cohesion becomes progressively weakened [4], predisposing the oocytes to greater risk of premature separation of sister chromatids (PSSC) during oocyte maturation [2-5] (Figure 1).

Given the great time lapse, it seems daunting that any measure can be found to keep these primary oocytes “fresh” while women age. Our study however suggests that a metabolic deficiency during ovulation in older females, which can be easily corrected, is an important compounding factor in age-related egg aneuploidies. It has been known for more than four decades [6] that during mammalian ovulation, the ovaries exhibit a transitory (i.e. a few hours) rise in the enzyme ornithine decarboxylase (ODC) and its product putrescine and yet its physiological function remains enigmatic. We found that inhibition of ODC during ovulation results in significant increase of incidence of PSSC in eggs of young mice. Interestingly, older mice exhibit a diminished ODC rise in the ovaries, and a corresponding increase of PSSC in eggs. Most remarkably, a combination of putrescine supplementation in mouse drinking water leading up to oocyte retrieval and in oocyte maturation medium reduced the incidence of PSSC in eggs by more than 50% [2].

Putrescine is a naturally occurring, small metabolite easily absorbed and distributed throughout the organism. A high dose of putrescine supplementation similar to that used in our study has been given safely to neonatal calves and pigs as an ingredient of soybean-based milk replacement [7]. However, post-conception putrescine supplementation in mouse drinking water compromises fetal development, likely due to excess reactive oxygen species produced by placental diamine oxidase oxidizing the exogenous putrescine [8]. In contrast, periovulatory putrescine supplementation is safe for gestation, because exogenous putrescine administered around ovulation is cleared long before placental development (Tao and Liu, in preparation). Nonetheless, further studies, including that employing primates, are required to formulate effective and safe putrescine supplementation protocols to reduce egg aneuploidies, both in IVF clinics and in natural conception.
